# Correlation between the severity of endometriosis and the occurrence of placenta accreta spectrum in the subsequent pregnancy

**DOI:** 10.12669/pjms.41.1.11145

**Published:** 2025-01

**Authors:** Zhenna Wang, Yi Xie, Yanhui Mao, Shihan Yan, Jingyu Huang, Shunhe Lin

**Affiliations:** 1 Zhenna Wang Department of Obstetrics and Gynecology, Fujian Maternity and Child Health Hospital, College of Clinical Medicine for Obstetrics & Gynecology Pediatrics, Fujian Medical University, Fuzhou, Fujian Province 350001, P.R. China; 2 Yi Xie Department of Obstetrics and Gynecology, Fujian Maternity and Child Health Hospital, College of Clinical Medicine for Obstetrics & Gynecology Pediatrics, Fujian Medical University, Fuzhou, Fujian Province 350001, P.R. China; 3 Yanhui Mao Department of Obstetrics and Gynecology, Fujian Maternity and Child Health Hospital, College of Clinical Medicine for Obstetrics & Gynecology Pediatrics, Fujian Medical University, Fuzhou, Fujian Province 350001, P.R. China; 4 Shihan Yan Department of Obstetrics and Gynecology, Fujian Maternity and Child Health Hospital, College of Clinical Medicine for Obstetrics & Gynecology Pediatrics, Fujian Medical University, Fuzhou, Fujian Province 350001, P.R. China; 5 Jingyu Huang Department of Obstetrics and Gynecology, Fujian Maternity and Child Health Hospital, College of Clinical Medicine for Obstetrics & Gynecology Pediatrics, Fujian Medical University, Fuzhou, Fujian Province 350001, P.R. China; 6 Shunhe Lin Department of Obstetrics and Gynecology, Fujian Maternity and Child Health Hospital, College of Clinical Medicine for Obstetrics & Gynecology Pediatrics, Fujian Medical University, Fuzhou, Fujian Province 350001, P.R. China

**Keywords:** Endometriosis, Placenta accreta spectrum, R-AFS score, Staging, Generalized additive modelling

## Abstract

**Objective:**

To investigate the correlation between endometriosis (EMs) severity and placenta accreta spectrum (PAS) risk in the subsequent pregnancy.

**Method:**

Clinical records of 2,142 patients who underwent laparoscopic surgery for EMs at Fujian Provincial Maternal and Child Health Hospital from January 2014 to January 2018, who had achieved pregnancy and were delivered, were analyzed. Baseline data, EMs stage, The Revised American Fertility Society (R-AFS) score, levels of serum indexes, and pregnancy and neonatal outcomes were recorded. The outcome of interest was the occurrence of PAS. The correlation between the R-AFS score, endometriosis staging, and PAS was explored, and the R-AFS threshold was identified.

**Results:**

PAS was associated with a higher incidence of chronic pelvic pain (OR = 8.68, 95% CI: 1.18-45.79, P = 0.014) and infertility for >five years (OR = 2.5, 95% CI: 1.35-4.65, P = 0.003), elevated serum levels of cancer antigens, higher incidence of postpartum hemorrhage, and placenta previa (P < 0.05). PAS rate was higher in women with higher EMs staging and R-AFS score and ovarian EMs combined with deep infiltrating endometriosis (DIE) (P < 0.001). After adjusting for confounders, both R-AFS score (AOR = 1.02, 95% CI: 1.01-1.03, P < 0.001) and ovarian EMs with DIE (AOR = 3.31, 95% CI: 1.54-6.67, P = 0.001) were independent risk factors for PAS. R-AFS score of 29 was identified as a threshold for an increased risk of PAS.

**Conclusion:**

The risk of PAS in patients with endometriosis increases with the R-AFS score. PAS is more likely to occur in women with ovarian EMs combined with DIE. It is necessary to implement a specific monitoring program during pregnancy in patients with a history of severe EMs

## INTRODUCTION

Endometriosis (EMs) is a common gynecological disease that may lead to dysmenorrhea, chronic pelvic pain, infertility, etc.^1^,^2^ About 10% of women of reproductive age are estimated to present varying degrees of EMs.^3^,^4^ Even after conception, EMs may increase the likelihood of obstetric complications, potentially resulting in adverse pregnancy outcomes.^5^ Women with EMs are more prone to have abnormal placenta position and placenta accreta spectrum (PAS) during pregnancy.^6^,^7^ PAS refers to a group of disorders characterized by varying degrees of placental villi invasion into the uterine myometrium and is considered a significant cause of severe postpartum hemorrhage, organ damage, preterm birth, and even maternal and fetal death.^7^,^8^ Predicting PAS before delivery and guiding the development of personalized prenatal care and delivery plans during pregnancy and labor is of practical significance for ensuring safe childbirth. History of cesarean section and placenta previa has been identified as an independent risk factor for PAS, and a history of uterine procedures and surgeries is considered a high-risk factor.^9^–^11^

However, these factors do not fully explain why PAS occurs in some primiparous women. Moreover, there is still a lack of understanding regarding the correlation between the severity of EMs and the strength of its association with PAS. The main objectives of this study were to investigate the potential association between the severity of EMs and PAS and to determine the risk threshold for developing PAS in the next pregnancy. These findings may help to develop a gestational maternity program for patients with a history of EMs and to screen high-risk pregnant women to improve the pre-delivery incidence of PAS.

## METHODS

Medical records of patients with EMs who underwent diagnostic laparoscopic surgery from January 2014 to January 2018 at Fujian Provincial Maternal and Child Health Hospital were selected. Patients were followed up for five years, and cases of recorded pregnancies and deliveries at 28 weeks postoperatively were recorded. Only the first pregnancy was taken as the study outcome for patients with more than one successful pregnancy and delivery within five years. Grouping was based on these pathological diagnostic criteria, as described in 1937 by Irving and Hertig.^12^ Patients with clinically suspected pathology and a diagnosis were retrospectively divided into the PAS and the non-PAS groups.

### Ethical Approval:

The study was approved by the Ethics Committee of Fujian Maternal and Child Health Hospital (2024KY046); Approval Dated: March 27^th^, 2024. Ethical approval and written informed consent were not required as the patients were not intervened at any stage and were only followed up for their natural pregnancy outcome.

### Inclusion criteria:

- Patients who underwent diagnostic laparoscopic surgery for EMS.
- Staging of Ems according to the American Society for Reproductive Medicine (R-AFS) method.^13^
- Patients who achieved at least one successful pregnancy and delivered after > 28 weeks of gestation.
- Transabdominal ultrasonography for PAS was done during the pregnancy.
- Complete medical information and follow-up of at least five years after the laparoscopy.

### Exclusion criteria:

- Loss to follow-up.
- Patients who did not achieve successful pregnancy.
- Previous history of PAS.
- Pregnancy terminated before 28 weeks of gestation.
- Incomplete clinical records.

### Diagnostic criteria and staging of EMs:

Laparoscopic surgery was followed by a pathological examination to confirm the diagnosis of EMs when typical EMs peritoneal implantation foci were seen during the surgery and/or when suspicious lesions/endocytic walls were excised (stripped). The staging was done according to the R-AFS method.^13^ R-AFS staging is mainly based on the size and depth of peritoneal and ovarian lesions, the extent and degree of ovarian and tubal adhesions, and the degree of closure of the recto-uterine trap. It is divided into four stages: Stage-I (tiny lesions): one to five points; Stage-II (mild): six to fifteen points; Stage-III (moderate): sixteen to forty points; and Stage-IV (severe): greater than or equal to forty points.

The diagnostic criteria of PAS were based on the final histopathological examination of the placenta, utilizing samples from uterine curettage, myometrial tissue, or postpartum placental tissue. Classification was conducted according to the clinical and histopathological criteria,^12^ enabling precise identification of PAS subtypes. Placenta accreta (PA) was diagnosed if the invasion depth was limited to the superficial myometrium; Placenta increta (PI) was diagnosed if the invasion extended into the deeper myometrium; and Placenta percreta (PP) was diagnosed if the invasion penetrated the entire uterine wall, reaching the uterine serosa or neighboring organs.^14^ Clinical information on the patients from the period of laparoscopic surgery and the period of pregnancy and childbirth was collected from the hospital’s medical record system. It included:

- General information (Age, BMI, gravidity and parity, history of previous cesarean section, history of previous uterine surgery, duration of infertility, etc.).
- Pregnancy and delivery information (gestational week at delivery, gestational diabetes, post-partum hemorrhage, placenta previa, placental abruption, placental adhesions, placental accreta, etc.).
- EMs classification and staging, including R-AFS score; 4) PAS classification; 5) Laboratory indexes including serum cancer antigens 19-9 (CA19-9), and 125 (CA-125), anti-mullerian hormone (AMH), etc.

### Statistical Analysis:

The analysis was performed using R software (version 4.2.2). Normally distributed measurements were expressed as mean (± standard deviation) (± s). The independent samples t-test was used for data that conformed to normal distribution. Count data were expressed as percentages (number of cases) (%, n). The x-squared test or Fisher’s exact probability method was used to compare groups. The correlation between R-AFS scores and PAS, and between the types of EMs and PAS, was further explored using a logistic regression model corrected for confounders, and adjusted odds ratios (ORs) and 95% confidence intervals (CIs) were reported. P < 0.05 was considered significant. A generalized additive model (GAM) model was used to fit a smoothed plot of the relationship between R-AFS and PAS and to identify the threshold.

## RESULTS

**Fig.1:**
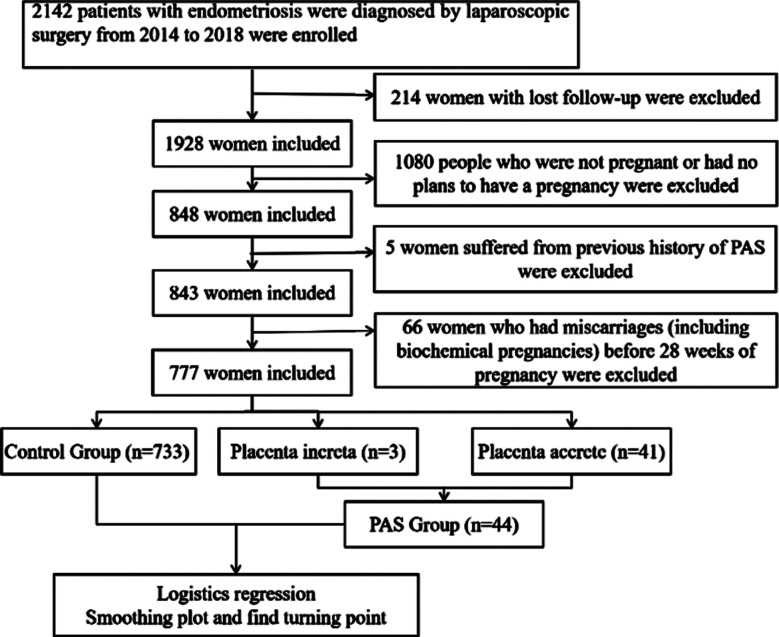
Flowchart of this study.

A total of 2,142 patients with EMs who underwent laparoscopic surgery and were diagnosed were screened from the hospital cohort. The flowchart of the study design is shown in Fig.1. Two hundred fourteen patients were excluded due to loss-to-follow-up, 1,080 patients were excluded as they had not conceived or had no plans to conceive, five patients were excluded due to a history of PAS, and sixty-six patients were excluded due to miscarriage before 28 weeks of gestation (including biochemical pregnancies). A total of 777 patients were included in the study, all successfully conceived and delivered at least once, with pregnancies reaching at least 28 weeks of gestation. Of these, 44 cases of PAS were identified. The PAS group included three cases of PI and 41 cases of PA. In this study, 44 women (5.7% of the total study population) developed PAS-related disorders during pregnancy.

The baseline characteristics of the included patients are presented in Table-I. Compared to the non-PAS group, the PAS group had a significantly higher prevalence of chronic pelvic pain (p=0.041) and a considerably higher incidence of infertility lasting more than five years (p=0.004). As shown in Table-II, the levels of tumor markers CA 125 and CA 199 were significantly higher in the PAS group (p<0.05). The median CA 125 level in the PSA group was 69.15 (interquartile range (IQR): 38.15, 126.08) compared to 43.7 (IQR: 27.6, 80.2) in the non-PAS group (p=0.025). For CA 199, the median level in the PAS group was 75.75 (IQR: 22.58, 117.95) compared to 32.38 (IQR: 15.58, 65.4) in the non-PAS group (p<0.001). In contrast, PAS was associated with significantly lower levels of AMH, with a median of 1.78 (IQR: 0.85, 3.94), compared to 2.45 (IQR: 1.34, 5.98) in the non-PAS group (p=0.034).

**Table-I:** General baseline information.

	Total (n=777)	Non-PAS (n=733)	PAS (n=44)	p value
Age (y)	31 (29, 34)	31 (29, 34)	30.5 (27, 34.25)	0.243
BMI (kg/m2)	20.94 (19.31, 22.85)	20.94 (19.37, 22.85)	20.77 (19.09, 22.45)	0.588
Parity, n(%)				0.698
0	502 (65)	475 (65)	27 (61)	
1	271 (35)	254 (35)	17 (39)	
2	4 (1)	4 (1)	0 (0)	
History of cesarean section, n(%)				1
0	725 (93)	684 (93)	41 (93)	
1	52 (7)	49 (7)	3 (7)	
History of uterine cavity operation, n(%)				0.183
0	349 (45)	334 (46)	15 (34)	
1	428 (55)	399 (54)	29 (66)	
Dyspareunia, n(%)				0.076
0	715 (92)	678 (92)	37 (84)	
1	62 (8)	55 (8)	7 (16)	
Dysmenorrhea, n(%)				0.117
0	450 (58)	430 (59)	20 (45)	
1	327 (42)	303 (41)	24 (55)	
Chronic pelvic pain, n(%)				0.041
0	771 (99)	729 (99)	42 (95)	
1	6 (1)	4 (1)	2 (5)	
Infertile for more than 5 years, n(%)				0.004
0	515 (66)	495 (68)	20 (45)	
1	262 (34)	238 (32)	24 (55)	
Uterine adhesions, n(%)				1
0	756 (97)	713 (97)	43 (98)	
Mild	19 (2)	18 (2)	1 (2)	
Moderate	1 (0)	1 (0)	0 (0)	
Severe	1 (0)	1 (0)	0 (0)	
Spontaneous conception, n(%)				0.506
0	238 (31)	227 (31)	11 (25)	
1	539 (69)	506 (69)	33 (75)	

PAS, Placenta accreta spectrum; BMI, Body mass index.

**Table-II:** Preoperative CA125/CA199 level and intraoperative exploration.

	Total (n=777)	Non-PAS (n=733)	PAS (n=44)	p value
CA125, (U/mL)	44.3 (27.6, 81.2)	43.7 (27.6, 80.2)	69.15 (38.15, 126.08)	0.025
CA199, (U/mL)	32.7 (15.89, 72.2)	32.28 (15.58, 65.4)	75.75 (22.58, 117.95)	<0.001
AMH, (ng/mL)	2.34 (1.34, 5.96)	2.45 (1.34, 5.98)	1.78 (0.85, 3.94)	0.034
Stage, n(%)				<0.001
3	501 (64)	485 (66)	16 (36)	
4	276 (36)	248 (34)	28 (64)	
Adenomyosis, n(%)				1
0	704 (91)	664 (91)	40 (91)	
1	73 (9)	69 (9)	4 (9)	
R-AFS	32 (25, 56)	32 (25, 54)	51 (38.75, 94.25)	<0.001
OE merged with DIE, n(%)				0.003
0	699 (90)	666 (91)	33 (75)	
1	78 (10)	67 (9)	11 (25)	

PAS, Placenta accreta spectrum; OE, Ovarian endometriosis; DIE, Deep infiltrating endometriosis; R-AFS: Revised American Fertility Society score.

Diagnosis of PAS was associated with a significantly higher incidence of stage IV EMs (64% vs. 34% in the non-PAS group, p<0.001). The R-AFS scores were also significantly higher in the PAS group, with a median score of 51 (IQR: 38.75, 94.25) compared to 32 (IQR: 25, 54) in the non-PAS group (p<0.001). Additionally, the occurrence of ovarian endometrioma combined with deep infiltrating endometriosis (OE+DIE) was more common in the PAS patients (25% vs. 9%, p=0.003). However, there was no significant association between PAS and adenomyosis (p>0.05). Table-III shows that the proportion of postpartum hemorrhage, placenta previa, placental abruption, placental adhesion, placenta accreta, and battledore placenta was significantly higher in the PAS group (p<0.01).

**Table-III:** Pregnancy complications.

	Total (n=777)	Non-PAS (n=733)	PAS (n=44)	p value
Delivery gestational week, (n)	39 (37.86, 40)	39 (37.86, 40)	38.86 (32.43, 39.71)	0.066
GDM, n(%)				0.448
0	593 (76)	562 (77)	31 (70)	
1	184 (24)	171 (23)	13 (30)	
Postpartum hemorrhage, n(%)				0.002
0	760 (98)	721 (98)	39 (89)	
1	17 (2)	12 (2)	5 (11)	
Placental abruption, n(%)				1
0	758 (98)	715 (98)	43 (98)	
1	19 (2)	18 (2)	1 (2)	
Placenta previa, n(%)				<0.001
0	735 (95)	702 (96)	33 (75)	
1	42 (5)	31 (4)	11 (25)	
Placenta accreta, n(%)				<0.001
0	736 (95)	733 (100)	3 (7)	
1	41 (5)	0 (0)	41 (93)	
Placenta increta, n(%)				<0.001
0	774 (100)	733 (100)	41 (93)	
1	3 (0)	0 (0)	3 (7)	
Battledore placenta, n(%)				0.02
0	766 (99)	725 (99)	41 (93)	
1	11 (1)	8 (1)	3 (7)	
Neonatal weight, (g)	3210 (2795, 3510)	3210 (2805, 3515)	3147.5 (1860, 3492.5)	0.154

PAS, Placenta accreta spectrum; GDM, Gestational diabetes mellitus.

**Fig.2:**
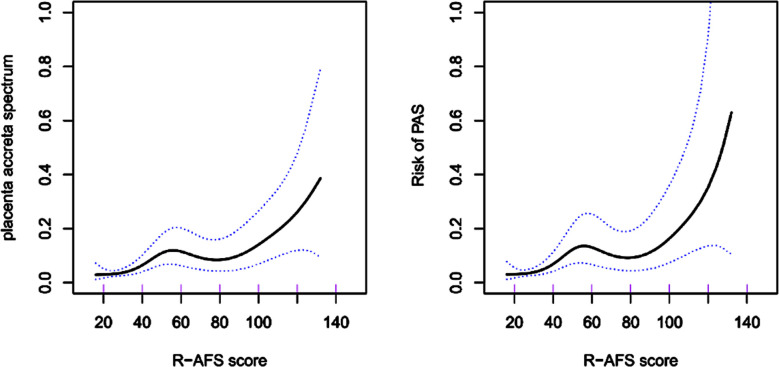
The relationship between r-AFS and PAS in pregnancy. (Horizontal coordinates of both graphs are r-AFS, vertical coordinates of the left graph are the probability of developing PAS, vertical coordinates of the right graph are the risk of developing PAS, the solid line in the middle is the fitted line and the dashed lines on either side are 95% confidence intervals).

Logistic models with PAS as the dependent variable and R-AFS score and OE+DIE as independent variables were generated and adjusted for confounding factors to estimate the AOR for the occurrence of PAS in women with higher R-AFS scores and OE+DIE. Based on the results of univariate analysis and clinical significance, variables such as age, BMI, infertility duration over five years, dysmenorrhea, history of cesarean section, history of uterine surgery, concurrent adenomyosis, and postoperative GnRHa treatment were gradually included in the model. After adjusting for relevant confounding factors (Models A-D), the results indicated that as the R-AFS scores increased, the risk of developing PAS during pregnancy also increased (AOR=1.02, 95% CI: 1.01-1.03). Additionally, OE+DIE was identified as another independent risk factor for PAS during pregnancy (AOR=3.31, 95%CI: 1.54-6.67) (Table-IV & V). The increase in the R-AFS scores was accompanied by the gradually increasing risk of PAS. Further analysis of the threshold effect identified an R-AFS score of 29 as a threshold (Fig.2).

**Table-IV:** Adjusted ORs for PAS according to R-AFS.

	Model A		Model B		Model C		Model D	

	AOR (95%CI)	P value	AOR (95%CI)	P value	AOR (95%CI)	P value	AOR (95%CI)	P value
R-AFS	1.02 [1.01, 1.03]	<0.001	1.02 [1.01, 1.03]	<0.001	1.03 [1.01, 1.04]	<0.001	0.05 [0.00, 1.86]	0.103
Age			0.97 [0.90, 1.05]	0.528	0.96 [0.88, 1.04]	0.358	1.02 [1.01, 1.04]	<0.001
BMI			0.95 [0.84, 1.07]	0.405	0.95 [0.83, 1.07]	0.396	0.97 [0.88, 1.05]	0.423
Infertile for more than 5 years					2.66 [1.39, 5.12]	0.003	0.95 [0.84, 1.07]	0.424
Dysmenorrhea					1.07 [0.51, 2.20]	0.865	2.72 [1.43, 5.25]	0.002
History of uterine cavity operation					2.12 [1.09, 4.30]	0.031	1.08 [0.52, 2.25]	0.826
History of cesarean section					1.04 [0.23, 3.29]	0.956	2.06 [1.06, 4.17]	0.039
Adenomyosis					0.58 [0.16, 1.61]	0.339	1.02 [0.23, 3.24]	0.977
postoperative treatment with GnRHa							0.57 [0.16, 1.59]	0.324

BMI, Body mass index; GnRHa, gonadotrophin-releasing; R-AFS: Revised American Fertility Society score.

**Table-V:** Adjusted ORs for PAS according to OE merged with DIE.

	Model A		Model B		Model C		Model D	

	AOR (95%CI)	P value	AOR (95%CI)	P value	AOR (95%CI)	P value	AOR (95%CI)	P value
OE merged with DIE	3.31 [1.54, 6.67]	0.001	3.38 [1.57, 6.83]	0.001	4.15 [1.86, 8.75]	<0.001	4.04 [1.80, 8.59]	<0.001
Age			0.96 [0.88, 1.04]	0.301	0.95 [0.87, 1.04]	0.257	0.96 [0.87, 1.04]	0.318
BMI			0.97 [0.86, 1.08]	0.595	0.97 [0.86, 1.10]	0.679	0.98 [0.86, 1.10]	0.716
Infertile for more than 5 years					3.01 [1.59, 5.78]	0.001	3.01 [1.58, 5.78]	0.001
Dysmenorrhea					1.97 [1.01, 3.88]	0.048	1.95 [1.00, 3.86]	0.052
History of uterine cavity operation					1.70 [0.89, 3.37]	0.115	1.68 [0.88, 3.34]	0.124
History of cesarean section					1.09 [0.24, 3.40]	0.900	1.02 [0.23, 3.23]	0.976
Adenomyosis					0.75 [0.21, 2.11]	0.621	0.68 [0.19, 1.93]	0.512
postoperative treatment with GnRHa							2.33 [1.02, 6.27]	0.064

OE, Ovarian endometriosis; DIE, Deep infiltrating endometriosis; BMI, Body mass index; GnRHa, Gonadotrophin releasing hormone agonist.

## DISCUSSION

This study found that the risk of developing PAS in women with previously diagnosed EMs significantly increases with higher preoperative r-AFS scores, showing a critical threshold effect. The results showed that PAS was more likely to occur in women with ovarian EMs combined with DIE.

There has been an increasing number of studies on the occurrence of pregnancy-related complications after endometriosis surgery in recent years, mainly focusing on the hypertensive disorders of pregnancy, postpartum hemorrhage, and placenta previa.^15^,^16^ At the same time, only a few studies assessed the impact of EMs on the spectrum of placental implantation, particularly the correlation between the severity of EMs and PAS. The prevalence of PAS is approximately 0.05-0.84% in the general population and is on the rise.^17^-^19^ This study showed that the prevalence of PAS was about 5% in the population with EMs, which was significantly higher than that in pregnant women without EMs, similar to the finding of Matsuaki et al.^20^

The results indicate that the R-AFS score and the presence of OE combined with DIE are independent risk factors for developing PAS in subsequent pregnancies and demonstrate that the R-AFS score has a threshold effect on the PAS risk. Vercellini et al.^21^ conducted a propensity-matched analysis of women who conceived naturally for the first time post-surgery based on the location of their EMs and found that DIE patients had nearly a six-fold increased risk of placental abnormalities (OR 5.81, 95% CI: 1.53–22.03), whereas no PAS cases were observed in women with OE. The junctional zone (JZ) is critical in normal uterine peristalsis.^22^ Structural and functional changes in the inner myometrium (or JZ) may lead to the failure of physiological transformation of the spiral arteries at the uteroplacental bed, thus affecting placental formation.^23^

Abnormalities in the JZ could result in altered endometrial receptivity and defects in trophoblast invasion or migration. Studies have shown that the JZ is enlarged in EMs patients, suggesting structural and functional abnormalities in the uterine wall.^24^ Notably, women with EMs have higher uterine contraction frequency, amplitude, and basal pressure than women without EMs.^25^ Disruption of uterine peristalsis may influence the final location of blastocyst implantation, thereby increasing the risk of placental abnormalities.^21^,^26^,^27^

The alterations in the endometrial microenvironment, in particular, immune equilibrium at the maternal-fetal interface in patients with EMs are considered a potential mechanism leading to PAS.^28^ One of the pathological features of PAS is the formation of thick fibrin-like deposits at the uteroplacental interface.^29^ Damage to the myometrium manifests as disordered muscle fibers, tissue edema, inflammation, and elastic degeneration, all of which can result in abnormal decidualization.^26^ This damage may cause extravillous trophoblasts to invade the radial and/or arcuate arteries, leading to high-velocity maternal blood flow entering the intervillous space. Patients with higher stages of EMs also exhibit greater reductions in NK cell activity.^30^

The imbalance in estradiol levels enhances tissue adhesiveness, increases the activity of matrix metalloproteinases, and triggers angiogenic responses.^31^ All these local changes in the endometrial environment in women with EMs increase their risks for preterm birth, fetal growth restriction, and placental disorders.^24^,^32^ Additionally, this study suggests that chronic pelvic pain, long (over five years) infertility, and elevated levels of CA 125 and CA 199 may be significant clinical indicators of increased PAS risk during pregnancy in patients with EMs. Chronic pelvic pain may be associated with the progression of EMs, leading to chronic intra-abdominal inflammation, along with adhesion, growth, angiogenesis, and fibrosis of endometrial tissue outside the uterus.^33^,^34^ These pathophysiological features result in chronic pelvic pain, potentially reflecting a more severe form of EMs. Similarly, a longer duration of infertility and elevated CA125 and CA199 levels may also correlate positively with the severity of EMs.^35^-^37^ These factors are likely associated with the extent and severity of the disease. Given the complexity of EMs subtypes, this study utilized the classic R-AFS score as the independent variable, which encompasses different EMs subtypes. This scoring system provides a comprehensive assessment of the disease’s impact, supporting the identification of patients at higher risk for PAS during pregnancy.^38^

This study has important clinical implications. Based on its observations, the ASRM score may be used to develop personalized maternity protocols for postoperative endometriosis pregnancies and to alert clinicians to the possibility of PAS. In this study, severe EMs were found to be associated with the occurrence of PAS in subsequent pregnancies, especially with EMs scores greater than 29 or more, with a significant increase in the odds of occurrence with each additional point. In addition, the common OE phenotype of EMs increases the risk of PAS if it is also comorbid with DIE. Therefore, during the real-time routine obstetric examination of pregnant women, the follow-up history should include careful enquiry about a history of previous surgeries, including EMs surgery, as well as the ASRM score and subtypes. For scores greater than 29 or a diagnosis of OE combined with DIE, the obstetric ultrasound examination should be strengthened during the obstetric examination to pay special attention to the placenta implantation and record any incidence of PAS to avoid missed diagnosis. Early detection of PAS before delivery and formulation of a detailed corresponding plan is beneficial for reducing postpartum hemorrhage and delivery complications.

### Limitations:

First, this was a retrospective cohort study with a potential risk of selection bias. For instance, since all patients with EMs who underwent laparoscopic surgery were likely in the later stages of the disease, patients diagnosed with stage I and II EMs might have been underrepresented. Additionally, the cohort in this study consisted exclusively of Asian Han women, which may limit the generalizability of the results to other ethnic groups. Furthermore, the study did not differentiate between postoperative treatment regimens, which could have influenced the outcomes. Furthermore, the ASRM scoring system for endometriosis involves subjective assessments, which can affect the study’s outcomes and conclusions. To compensate for this potential subjectivity, this study used OE combined with DIE as the dependent variable and confirmed a correlation between endometriosis severity and the spectrum of placental implantation. Additionally, although there was a correlation between the staging and typing of EMs and the risk of PAS, the threshold effect of the R-AFS score on PAS risk requires further validation in multicenter studies with larger sample sizes.

These limitations suggest that while this study provides valuable insights into the relationship between EMs and PAS, the results should be interpreted with caution. Further research is needed to confirm the observations and extend these results across diverse populations and clinical settings.

## CONCLUSION

Higher staging of EMs and the presence of ovarian endometrioma combined with deep infiltrating endometriosis are associated with an increased risk of PAS during pregnancy. This study identified a specific risk threshold for the R-AFS score. It confirmed that targeted monitoring protocols are needed for pregnant women with severe EMs, particularly in cases of an R-AFS score exceeding 29 or OE combined with DIE. This proactive approach may help mitigate the risk of PAS and improve maternal and fetal outcomes.

### Authors’ Contributions:

**ZW, YX and YM:** Conception and design.

**YM, JH and SY:** Provision of study materials, patients.

**YX, YM and JH:** Collection of data.

**SL and SY:** Data analysis and interpretation.

**ZW, YX and YM:** Manuscript writing.

All authors have read the final version, approved it and are also responsible for the clinical integrity of the study.
